# Age-dependent increase in antibodies that inhibit *Plasmodium falciparum* adhesion to a subset of endothelial receptors

**DOI:** 10.1186/s12936-019-2764-4

**Published:** 2019-04-11

**Authors:** Oumar Attaher, Almahamoudou Mahamar, Bruce Swihart, Amadou Barry, Bacary S. Diarra, Moussa B. Kanoute, Adama B. Dembele, Sekouba Keita, Santara Gaoussou, Djibrilla Issiaka, Alassane Dicko, Patrick E. Duffy, Michal Fried

**Affiliations:** 1 Malaria Research & Training Center, Faculty of Medicine, Pharmacy and Dentistry, University of Sciences Techniques and Technologies of Bamako, P.O Box 1805, Bamako, Mali; 2 Biostatistics Research Branch, National Institute of Allergy and Infectious Diseases, NIH, Rockville, MD USA; 3 Laboratory of Malaria Immunology and Vaccinology, National Institute of Allergy and Infectious Diseases, NIH, Rockville, MD USA

**Keywords:** Sequestration, Anti-adhesion antibodies, IE (infected erythrocytes) surface proteins

## Abstract

**Background:**

*Plasmodium falciparum*-infected erythrocytes (IE) sequester in deep vascular beds where their adhesion is mediated by an array of endothelial surface receptors. Because parasite adhesion has been associated with disease, antibodies that block this activity may confer protective immunity. Here, levels of plasma anti-adhesion activity and surface reactivity against freshly collected IEs from malaria-infected children were measured in a Malian birth cohort and related to child age and malaria infection history.

**Methods:**

Plasma samples from children enrolled at birth in a longitudinal cohort study of mother–infant pairs in Ouelessebougou, Mali were collected at multiple time points during follow-up visits. Anti-adhesion antibodies (i.e., inhibit IE binding to any of several endothelial receptors) and reactivity with surface IE proteins were measured using a binding inhibition assay and by flow cytometry, respectively.

**Results:**

Levels of antibodies that inhibit the binding of children’s IE to the receptors ICAM-1, integrin α_3_β_1_ and laminin increased with age. The breadth of antibodies that inhibit ICAM-1 and laminin adhesion (defined as the proportion of IE isolates whose binding was reduced by ≥ 50%) also significantly increased with age. The number of malaria infections prior to plasma collection was associated with levels of plasma reactivity to IE surface proteins, but not levels of anti-adhesion activity.

**Conclusions:**

Age is associated with increased levels of antibodies that reduce adhesion of children’s IE to three of the ten endothelial receptors evaluated here. These results suggest that anti-adhesion antibodies to some but not all endothelial receptors are acquired during the first few years of life.

**Electronic supplementary material:**

The online version of this article (10.1186/s12936-019-2764-4) contains supplementary material, which is available to authorized users.

## Background

Among the five human malaria parasites, *Plasmodium falciparum* is able to sequester in deep vascular beds of various tissues. Infected erythrocytes (IE) have been demonstrated to bind a number of receptors expressed on the endothelial cell surface, including thrombospondin (TSP), CD36, intercellular adhesion molecule-1 (ICAM-1), vascular cell adhesion molecule-1 (VCAM-1), E-selectin (ELAM-1), P-selectin, PECAM-1/CD31, EPCR, members of integrin family, extracellular matrix proteins like laminin and cellular fibronectin [1–13]. During pregnancy malaria, IEs bind chondroitin sulfate A (CSA) on the surface of the syncytiotrophoblast, the cellular syncytium that covers the placental villi [14]. The variant IE surface protein called PfEMP1 has been implicated in adhesion to several endothelial receptors as well as in antigenic variation, and is thus believed to play a key role in severe disease due to *P. falciparum* (reviewed in [15, 16]). Any single parasite appears to express a single PfEMP1 variant or more on the IE surface, beginning approximately 18 h into the erythrocytic phase of the parasite lifecycle [17–19], but expression can switch at the next cycle of invasion [20, 21]. PfEMP1 variants are encoded by approximately 60 *var* genes per haploid genome of *P. falciparum,* and display extensive variation within and between genomes (reviewed in [22]).

In nonhuman primate studies, treatment of malaria-infected animals with antibodies developed against the infecting parasite (cloned in another animal) rapidly reversed IE cytoadhesion, resulting in the release of the parasite’s mature forms into the peripheral blood circulation [23]. The same antibodies also inhibited parasite adhesion to melanoma cells in vitro [24]. Similarly, IgG purified from sera of immune west African adults effectively treats West African children [25] as well as Thai adults suffering from symptomatic malaria [26]. Among pregnant women, acquisition of antibodies to IE surface proteins that block parasite adhesion have been associated with improved outcomes, including reduction in infection, parasite density, increased birthweight, gestational age and maternal haemoglobin levels [27–31]. Previous studies from areas of stable malaria transmission reported that antibody levels to surface IE proteins are low in children aged 6–36 months [32, 33], and levels increase with age [33–35]. In children older than 6 years, antibody levels have been associated with protection from clinical malaria [35]. Similarly, age was also associated with increased IE agglutination activity [36].

Here, in the context of a longitudinal birth cohort, antibodies that inhibit IE adhesion to several endothelial receptors (anti-adhesion antibodies), and antibodies reacting with IE surface proteins of fresh parasites, were related with child age and prior malaria infection. The study was designed to evaluate plasma antibody to diverse surface proteins by assaying IE collected from children in the same cohort, described here as heterologous parasites.

## Methods

### Study population and clinical procedures

Evaluation of samples collected during a longitudinal cohort study of newborns and children aged 0–3 years conducted in Ouelessebougou, Mali. The study site is located 80 km south of Bamako, an area of intense but highly seasonal malaria transmission. Prior to enrollment, written informed consent was obtained from the parents/guardians on behalf of their children after receiving a study explanation form and oral explanation from a study clinician in their native language. The protocol and study procedures were approved by the institutional review board of the National Institute of Allergy and Infectious Diseases at the US National Institutes of Health (ClinicalTrials.gov ID NCT01168271), and the Ethics Committee of the Faculty of Medicine, Pharmacy and Dentistry at the University of Bamako, Mali. An intensive follow-up included monthly scheduled clinic visits during the malaria transmission season, and every 2 months during the dry season, as well as ad hoc visits when symptoms occurred. Clinical information was collected by project clinicians on standardized forms. Malaria infections were treated with artemether–lumefantrine or quinine as clinically indicated. Severe malaria was defined as parasitaemia detected by blood smear microscopy and at least one of the following World Health Organization criteria for severe malaria: > 2 convulsions in the past 24 h; prostration (inability to sit unaided or in younger infants inability to move/feed); haemoglobin < 5 g/dl; respiratory distress (hyperventilation with deep breathing, intercostal recessions and/or irregular breathing); coma (Blantyre score < 3). The presence of severe malaria symptoms due to other diseases were clinically ruled out.

### Parasite samples

Blood samples collected from malaria-infected children were used as the source of *P. falciparum* parasites in assays. Of 390 parasite-infected samples assayed, 15 samples were collected from children presenting with severe malaria. Parasite samples were collected from children aged (median (range)) 27.6 months (1.3–59.8 months), with parasite density of (median (range)) 72,325 parasites/µl (3250–566,025/µl). Each parasite sample was used for assays of 12–18 plasma samples, and plasma samples were assayed against an average (range) of 2.7 (1–9) parasite samples.

### Binding inhibition assay

Ring stage parasite samples were allowed to mature to the trophozoite/schizont stages during in vitro culture for 16–24 h, then mature parasite forms were enriched by gelatin flotation. Parasite binding inhibition assay was performed using a parasite suspension at 2–20% parasitaemia and 0.5% haematocrit [29]. 20 µl of recombinant endothelial receptors (CD36, ICAM-1, PECAM-1, P-selectin, integrin α_3_β_1_, integrin α_5_β_1_, integrin α_v_β_3_, JAM-B, from R&D Systems Minneapolis, MN, cellular fibronectin, and laminin from placenta from Sigma, St. Louis, MO) at a concentration of 10 µg/ml were immobilized by adsorption on Petri dishes. The parasite suspension was preincubated with plasma diluted 1:5 for 30 min at room temperature then allowed to bind to the immobilized receptor for 30 min at room temperature. Plates were washed three times with PBS to remove unbound cells. Bound cells were fixed in 0.5% glutaraldehyde for 10 min, stained with 10% Giemsa for 2 min and quantified by counting the number of parasites in 20 high-power fields. Level of inhibition was defined based on the level of IE binding in the presence of a pool of plasma samples collected from malaria-naïve adults in the USA (negative control) as follow (100 − (N_test_/N_control_) × 100)). A minimum IE count of 20 in the presence of naïve plasma was required for the results to be accepted.

### Flow cytometry

Mature IE were incubated with plasma samples diluted 1:10 followed by incubation with anti-human IgG conjugated to PE (eBioscience) and stained with SYBR green. A pool of hyperimmune adult plasma was used as a positive control and a pool of plasma samples collected from malaria-naïve adults was used as a negative control. Reactivity of antibodies with the IE surface is expressed as a ratio of the proportion of IE recognized by test and a pool of naïve plasma donors, as follows: Ratio = %IE_test_/%IE_control_.

### Statistical analysis

Data were collected on standardized forms and optically scanned into the database using DataFax (version 4.2, Clinical DataFax Systems, Inc., Hamilton, Ontario, Canada). Linear mixed effects models accounting for multiple measurements per child were fitted to relate anti-adhesion antibodies and reactivity with IE surface with age and number of previous infections. Breadth of anti-adhesion antibody activity and surface IE recognition were analysed using mixed effects beta binomial logistic regression models, accounting for the number of parasite isolates tested. Separate models were fitted for age and the number of previous malaria infections. P value was corrected for multiple comparisons using the Holm method. Analyses were carried out in R (version 3.3.2).

## Results

### Study population

Anti-adhesion and IE surface-reactive antibodies were measured in plasma samples collected from Malian children participating in longitudinal cohort studies. Depending on the endothelial receptor under study, anti-adhesion assays were performed with sera from 82 to 166 children at a median age of 17.8–28.6 months, and surface recognition by flow cytometry with sera from 106 children at a median age of 18.4 months. Many of the cohort children provided multiple plasma samples used in these assays, and typically had multiple *P. falciparum* infections documented before sample collection (Table 1). 82% of the children included here were blood smear-negative at the time of sample collection. The assays were performed using 390 parasite isolates including 15 samples collected from children who presented with severe malaria.

**Table 1:** Study population providing plasma samples

Receptor	n	Number of plasma samples/child Mean (range)	Median age (months)^a^	Number of previous infections Mean (SD)^a^
CD36	129	1.6 (1–3)	21.0	3.3 (2.6)
Cell. Fibronectin	105	1.9 (1–2)	18.3	2.9 (2.0)
ICAM-1	137	1.4 (1–3)	14.7	2.8 (2.1)
Integrin α_v_β_3_	110	1.7 (1–4)	22.0	2.7 (2.1)
Integrin α_3_β_1_	120	1.8 (1–5)	24.2	2.6 (1.9)
Integrin α_5_β_1_	129	2.1 (1–4)	28.6	2.8 (2.2)
JAM-B	138	2.0 (1–3)	17.8	2.7 (1.9)
Laminin	112	1.9 (1–4)	18.4	2.7 (2.1)
PECAM-1	166	1.9 (1–4)	18.3	2.7 (2.0)
P-selectin	82	1.3 (1–3)	23.8	2.4 (1.8)
IE surface (flow)	106	2.0 (1–2)	18.4	2.8 (2.0)

^a^For children providing multiple samples, age and number of previous infections at each time point were included

### Plasma inhibition of parasite binding increases with age

The level of anti-adhesion activity and the breadth of anti-adhesion activity were analysed in relation to age and number of prior infections. Plasma samples were tested for inhibiting IE adhesion to the following receptors: CD36, ICAM-1, P-selectin, platelet endothelial cell adhesion molecule-1 (PECAM-1/CD31), integrin α_3_β_1_, integrin α_5_β_1_, integrin α_v_β_3_, laminin, cellular fibronectin and JAM-B. This set of receptors includes molecules that in earlier studies, were shown to support IE adhesion to the endothelium, as well as novel receptors (integrin α_3_β_1_, laminin, cellular fibronectin and JAM-B) identified in a binding phenotype survey of IE collected from children in this study [13]. Level of binding to each receptor (IE counts) in the presence of plasma from naïve donors is shown in Additional file 1: Table S1. In recent years, severe malaria was associated with IE adhesion to the receptor EPCR [12]. In the current study, IE adhesion to EPCR was rare which did not allow evaluating acquisition of antibodies that block IE adhesion to this receptor. The assays were performed on fresh heterologous parasite isolates collected from malaria-infected children participating in the same longitudinal study as the children who provided plasma for antibody studies.

**Fig. 1:**
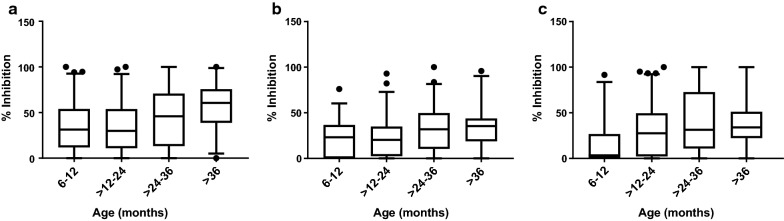
Binding inhibition levels stratified by age to the receptors ICAM-1 (**a**), integrin α_3_β_1_ (**b**), and laminin (**c**). Box plot indicates the median (horizontal line) and interquartile range (box), the whiskers indicate the 5th and 95th percentiles. Number of samples and statistical analysis are shown in Tables 1 and 2

Mixed effect models were fitted to relate anti-adhesion antibodies to the child’s age and number of previous infections. Anti-adhesion antibodies that block IE binding to the receptors ICAM-1, integrin α_3_β_1_ and laminin were positively associated with age (Table 2, Fig. 1). Anti-adhesion antibodies that block IE binding to the receptors P-selectin, PECAM-1, integrin α_5_β_1_, integrin α_v_β_3_, JAM-B and cellular fibronectin increased with age to a lesser degree and the change with age did not achieve significance (Table 2, Additional file 2: Figure S1).

**Table 2:** Association between age and antibodies that inhibit IE adhesion to endothelial receptors

Receptor	Coefficient (95% CI)	P value*
Level of anti-adhesion antibodies and age		
CD36	− 0.03 (− 0.32 to 0.26)	NS
Cell. Fibronectin	0.54 (0.06 to 1.02)	NS
ICAM-1	0.69 (0.37 to 1.02)	0.0003
Integrin α_v_β_3_	0.08 (− 0.24 to 0.40)	NS
Integrin α_3_β_1_	0.47 (0.18 to 0.76)	0.01
Integrin α_5_β_1_	0.13 (− 0.15 to 0.40)	NS
JAM-B	0.23 (− 0.16 to 0.61)	NS
Laminin	0.68 (0.29 to 1.08)	0.007
PECAM-1	0.32 (− 0.03 to 0.68)	NS
P-selectin	0.31 (− 0.01 to 0.64)	NS
Breadth of anti-adhesion antibodies and age		
CD36	− 0.002 (− 0.04 to 0.03)	NS
Cell. Fibronectin	0.035 (0.002 to 0.07)	NS
ICAM-1	0.037 (0.02 to 0.06)	0.01
Integrin α_v_β_3_	− 0.0005 (− 0.02 to 0.02)	NS
Integrin α_3_β_1_	0.015 (− 0.007 to 0.04)	NS
Integrin α_5_β_1_	0.004 (− 0.01 to 0.02)	NS
JAM-B	0.017 (− 003 to 0.04)	NS
Laminin	0.035 (0.02 to 0.05)	0.004
PECAM-1	0.025 (0.003 to 0.050	NS
P-selectin	0.019 (− 0.005 to 0.04)	NS

*NS* not significant

* Holm corrected P value. Models adjusted for hemoglobin type (HbAA, HbAC, HbAS)

The relationship between age and the breadth of anti-adhesion activity was further examined. For this analysis, a cut-off of > 50% inhibition was used as the threshold to define anti-adhesion activity [37]. As shown in Table 2, age was associated with a significant increase of the odds of inhibiting an isolate binding at a level of ≥ 50% to the receptors ICAM-1 and laminin. For each year of life, the odds of inhibiting IE binding of any isolate at a level of ≥ 50% to ICAM-1 and laminin increased by 57% and 51%, respectively. Number of prior infections was not associated with level or breadth of anti-adhesion antibodies (Additional file 1).

### Antibodies to the IE surface increase with the number of previous infections

Heterologous parasite isolates were also used to examine prospectively the association between age and number of previous infections and reactivity with IE surface proteins. On average, children providing plasma for this analysis had experienced 2.8 malaria infections from birth up to sample collection. Because clinical isolates typically contain multiple variant parasite forms [38], IE surface recognition was defined as the ratio of the proportion of IE recognized by the child’s plasma to the proportion of IE recognized by plasma from naïve donors (background reactivity). The number of prior malaria infections was positively associated with plasma reactivity to IE surface proteins (Table 3). To examine the relationship between the number of prior malaria infections and the breadth of IE surface recognition, a cut-off ratio (%IE_test_/%IE_control_) based on the median (1.4) was used to define positive recognition of a parasite isolate. The breadth of reactivity with IE surface proteins trended towards a positive association with the number of prior malaria infections, but the relationship did not achieve statistical significance (Table 3). Child age was not associated with increased levels or breadth of IE surface protein recognition.

**Table 3:** Relationships between antibodies to IE surface and prior malaria infections

	Coefficient (95% CI)	P value
Level of antibodies to IE surface proteins and number of prior infections	0.072 (0.009 to 0.134)	0.02
Breadth of antibodies to IE surface proteins and number of prior infections	0.048 (− 0.007 to 0.103)	0.09

## Discussion

Over years of exposure, residents of endemic areas acquire immunity that prevents severe disease and limits blood stage parasite growth [39, 40]. Extensive antigenic variation in proteins expressed on the IE surface have been implicated as a major factor for the slow development of immunity. *Var* genes have been classified into 3 major groups and 2 intermediate groups (A, B, C, B/A and B/C) based on homology in the flanking region and chromosome location [41]. Multiple studies reported increased transcript levels of group A and B/A *var* genes in children with severe malaria [16]. Further sequence analysis identified conserved tandem domains named domain cassettes (DCs) [42], with DC8 and DC13 expressed at higher levels in parasites associated with severe disease [43–45]. Analyses of antibody responses to large PfEMP1 repertoires showed age dependent increase in antibody levels, seropositivity and breadth [46, 47], with a subset of group A *var* genes (Group 2) more readily recognized by infant’s plasma than other PfEMP1s [46]. Antibody levels to recombinant CIDR domains of DC8 and DC13 of group A and B also increased with age more rapidly than antibody levels to recombinant domains associated with adhesion to the receptor CD36 [48, 49].

Infection induces variant-specific agglutinating antibodies and antibodies that recognize the IE surface by flow cytometry [50, 51]. Some parasite isolates are more readily recognized by children’s IgG, especially those collected from young children and children with severe malaria [50, 52] similar to the ordered recognition of PfEMP1 domains. As adults acquire antibodies that recognize surface IE, they also exhibit heterologous anti-adhesion activity against the majority of the parasite isolates, but children can only block IE adhesion of a limited number of isolates [24, 53]. Because falciparum parasite sequestration has been associated with disease, antibodies that block parasite adhesion may be critical for developing protective immunity [23, 24].

In the current study, the development of anti-adhesion antibodies to several endothelial receptors with fresh isolates collected from children participating in the same longitudinal cohort was examined. The two most common endothelial receptors for adhesion of IE collected from children in this cohort were CD36 and integrin α_v_β_3_ [13]. Neither age nor the number of infections was associated with increased antibody levels that inhibit IE adhesion to the most common endothelial receptors. Age was associated with increased levels of anti-adhesion antibody and breadth of inhibiting IE binding to the receptors ICAM-1, integrin α_3_β_1_ and laminin. These results suggest that it may take longer to acquire anti-adhesion antibodies to common receptors like CD36 and integrin α_v_β_3_, consistent with a previous report describing low levels of anti-adhesion antibodies to CD36 in children aged 3–4 years compared to children aged 10–11 years [33]. In the current study, children were followed up to the age of 5 years, thus antibody reactivity that develops over time (like anti-adhesion antibodies to CD36) may not be observed in this group of children.

Although levels or breadth of anti-adhesion antibodies was not significantly associated with the number of prior malaria infections after correcting for multiple comparisons, antibody levels and breadth of antibodies that inhibit IE binding to the receptor laminin were associated with an increased number of prior malaria infections before correction. Similarly, a trend for prior malaria infection to be associated with breadth of anti-adhesion antibodies to the receptors ICAM-1 and JAM-B was not statistically significant (Additional file 1: Table S2).

Infected erythrocytes surface recognition was associated with the number of prior malaria infections but not with child age. Plasma samples reacting with IE surface proteins may or may not contain antibodies with anti-adhesion activity, suggesting that the targets of these antibodies may not completely overlap [37]. Differences in the epitopes/antigens recognized by anti-adhesion versus surface-reactive antibodies may account for the different host factors related to acquisition of each of these antibody types.

Antibodies that block IE adhesion to any of the receptors tested here and antibodies reacting with IE surface proteins did not predict a reduction in the risk of severe malaria. This could be related to the age of the participants. In this population, the rate of severe malaria remains stable in children from ages 12–60 months [13].

## Conclusion

In summary, levels of antibodies that inhibit adhesion of freshly collected parasites to the receptors ICAM-1, integrin α_3_β_1_, and laminin increased with age. Similarly, the breadth of antibodies that inhibit adhesion to the receptors ICAM-1 and laminin increased with age. However, age was not associated with increased anti-adhesion activity to other receptors evaluated here. These results in an area of Mali with highly seasonal intense malaria transmission suggest that it may take several years to acquire functional antibodies that can inhibit IE adhesion to a wide array of endothelial receptors.

## Additional files

**Additional file 1: Table S1.** IE counts in the presence of naïve plasma sample. **Table S2.** Relationships between anti-adhesion antibodies and prior malaria infections.

**Additional file 2: Figure S1.** Binding inhibition levels stratified by age to the receptors CD36, C. fibronectin, integrin α_1_β_3_, integrin α_3_β_1_, JAM-B, PECAM-1 and P-selectin. Box plot indicates the median (horizontal line) and interquartile range (box), the whiskers indicate the 5th and 95th percentiles.

## Abbreviations

IE: infected erythrocytes
ICAM-1: intercellular adhesion molecule-1
