# Supplementary material for: Age-dependent increase in antibodies that inhibit Plasmodium falciparum adhesion to a subset of endothelial receptors
Source: Malar J. 2019 Apr 11;18:128. doi: 10.1186/s12936-019-2764-4 (PMC6458601; doi:10.1186/s12936-019-2764-4)
Supplement: Supplementary file 1 — Additional file 1: Table S1. IE counts in the presence of naïve plasma sample. Table S2. Relationships between anti-adhesion antibodies and prior malaria infections. [file 12936_2019_2764_MOESM1_ESM.docx]

Additional file 1:

Table S1. IE binding in the presence of naïve plasma sample

| Receptor | Mean binding (range) |
| --- | --- |
| CD36 | 319 (26-803) |
| C. fibronectin | 74 (26-274) |
| ICAM-1 | 125 (25-713) |
| Integrin αvβ3 | 80 (28-225) |
| Integrin α3β1 | 92 (25-972) |
| Integrin α5β1 | 76 (23-510) |
| JAM-B | 103 (21-760) |
| Laminin | 55 (26-128) |
| PECAM-1 | 72 (21-265) |
| P-selectin | 90 (24-391) |

Table S2. Relationships between anti-adhesion antibodies and prior malaria infections

| Level of anti-adhesion antibodies and number of prior infections | | | |
| --- | --- | --- | --- |
| Receptor | Coefficient (95% CI) | P value | Holm corrected P value |
| CD36 | 0.35 (-0.64-1.34) | NS | NS |
| Cell. Fibronectin | 0.61 (-1.17-2.39) | NS | NS |
| ICAM-1 | 1.48 (-0.63-3.59) | NS | NS |
| Integrin α_v_β_3_ | -1.60 (0.-3.48-0.29) | NS | NS |
| Integrin α_3_β_1_ | -0.81 (0.-2.74-1.13) | NS | NS |
| Integrin α_5_β_1_ | 0.87 (-0.63-2.37) | NS | NS |
| JAM-B | 1.09 (-0.49-2.67) | NS | NS |
| Laminin | 2.27 (0.04-4.50) | 0.045 | NS |
| PECAM-1 | 1.07 (-0.65-2.79) | NS | NS |
| P-selectin | -0.48 (-2.68-1.73) | NS | NS |
|  | | | |
| Breadth of anti-adhesion antibodies and number of prior infections | | | |
| Receptor | Coefficient (95% CI) | P value | Holm corrected P value |
| CD36 | 0.07 (-0.03-0.17) | NS | NS |
| Cell. Fibronectin | 0.08 (-0.04-0.21) | NS | NS |
| ICAM-1 | 0.12 (-0.02-0.26) | 0.09 | NS |
| Integrin α_v_β_3_ | -0.16 (-0.31—0.02) | 0.03 | NS |
| Integrin α_3_β_1_ | -0.04 (-0.19-0.11) | NS | NS |
| Integrin α_5_β_1_ | 0.05 (-0.05-0.14) | NS | NS |
| JAM-B | 0.07 (-0.005-0.15) | 0.07 | NS |
| Laminin | 0.15 (-0.001-0.29) | 0.05 | NS |
| PECAM-1 | 0.08 (-0.01-0.17) | NS | NS |
| P-selectin | 0.04 (-0.12-0.19) | NS | NS |
