# Supplementary material for: Age-dependent increase in antibodies that inhibit Plasmodium falciparum adhesion to a subset of endothelial receptors
Source: Malar J. 2019 Apr 11;18:128. doi: 10.1186/s12936-019-2764-4 (PMC6458601; doi:10.1186/s12936-019-2764-4)
Supplement: Supplementary file 2 — Additional file 2: Figure S1. Binding inhibition levels stratified by age to the receptors CD36, C. fibronectin, integrin α1β3, integrin α3β1, JAM-B, PECAM-1 and P-selectin. Box plot indicates the median (horizontal line) and interquartile range (box), the whiskers indicate the 5th and 95th percentiles. [file 12936_2019_2764_MOESM2_ESM.pdf]

Figure S1: Binding inhibition levels stratified by age. Box plot indicates the median (horizontal line) and interquartile range (box), the whiskers indicate the 5th and 95th percentiles. Number of samples and statistical analysis are shown in Tables 1 and 2.

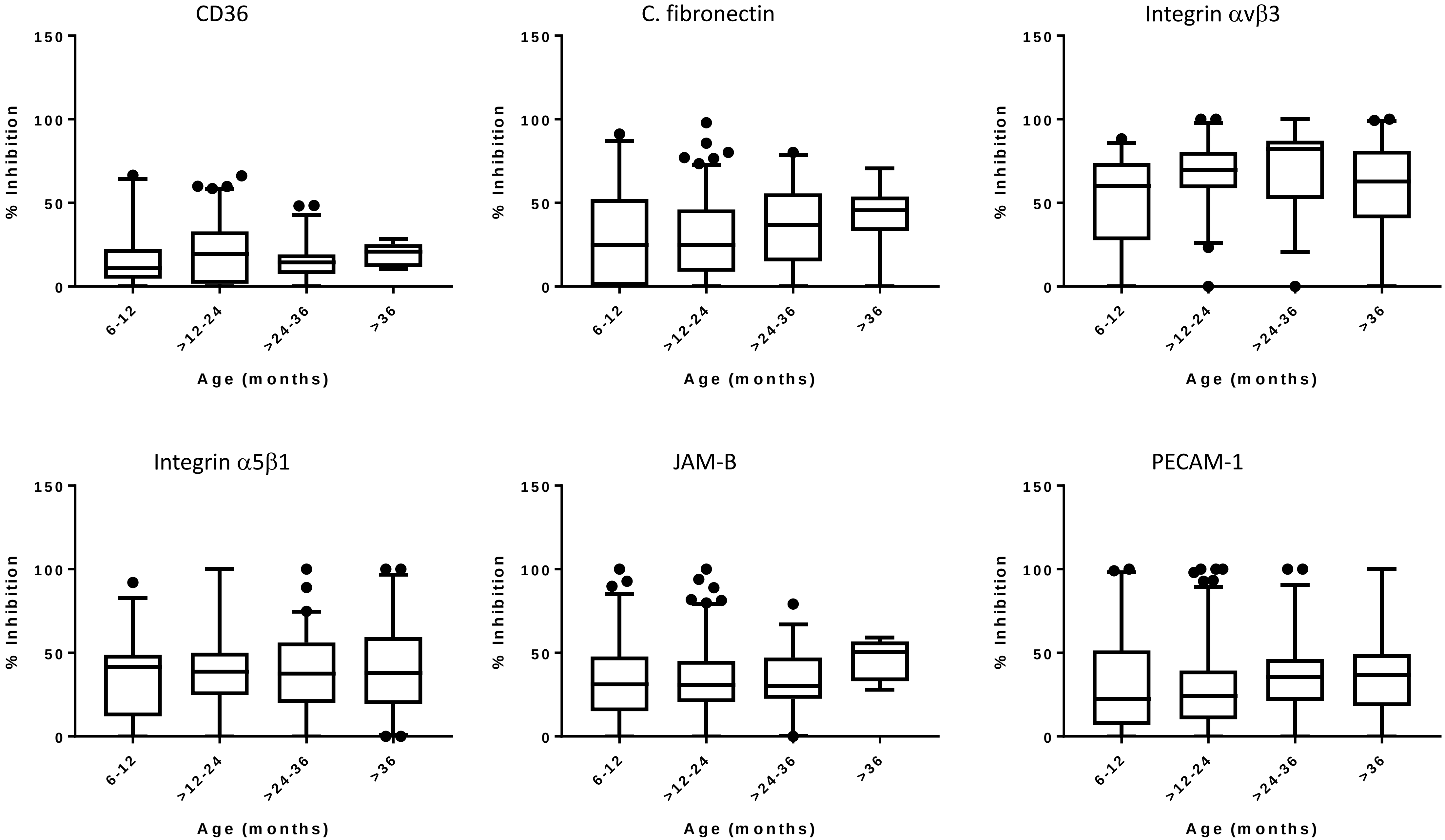

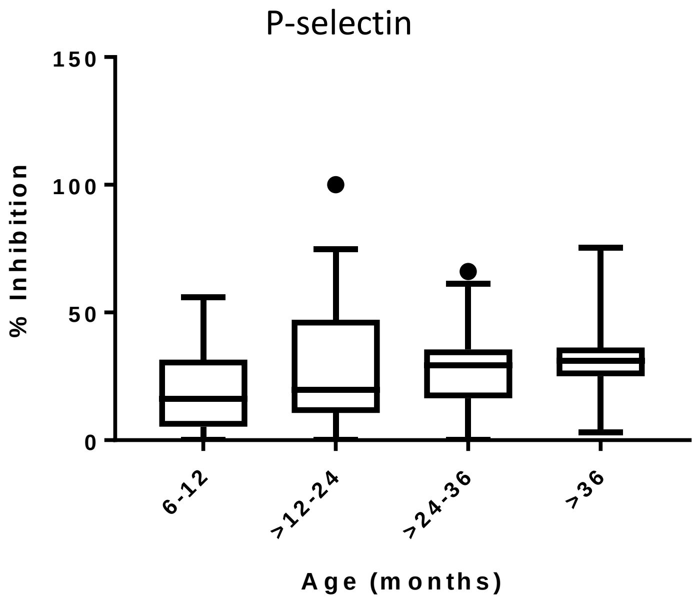
P-selectin
150
100
50
0
% Inhibition
6-12
>12-24
>24-36
>36
Age (months)
